# Summer Weight Gain Among Preschool-Aged Children With Obesity: An Observational Study in Head Start

**DOI:** 10.5888/pcd18.200532

**Published:** 2021-03-18

**Authors:** Jacob P. Beckerman-Hsu, Alyssa Aftosmes-Tobio, Kindra Lansburg, Jessie Leonard, Merieka Torrico, Erica L. Kenney, SV Subramanian, Sebastien Haneuse, Kirsten K. Davison

**Affiliations:** 1 Boston College School of Social Work, Chestnut Hill, Massachusetts; 2 Action for Boston Community Development, Boston, Massachusetts; 3 Community Action Agency of Somerville, Somerville, Massachusetts; 4 Department of Nutrition and Department of Social and Behavioral Sciences, Harvard T.H. Chan School of Public Health, Boston, Massachusetts; 5 Department of Social and Behavioral Sciences, Harvard T.H. Chan School of Public Health, Boston, Massachusetts; 6 Harvard Center for Population and Development Studies, Cambridge, Massachusetts; 7 Department of Biostatistics, Harvard T.H. Chan School of Public Health, Boston, Massachusetts

## Abstract

School-aged children gain weight most rapidly in summer, but few studies have investigated summer weight gain among preschool-aged children. We fit continuous linear spline mixed models to test for accelerated summer weight gain among 2,044 children attending 16 Boston-area Head Start programs between fall 2016 and spring 2019. Academic year and summer rates of change in modified body mass index z-score differed (*P* < .001), with accelerated summer weight gain most pronounced among children with obesity. As with school-aged children, increased focus on the summer is warranted for promoting healthy weight among children in Head Start.

**Summary**

**What is already known on this topic?**

School-aged children gain weight more rapidly in the summer than during the academic year. The limited evidence on summer weight gain among preschool-aged children is mixed.

**What is added by this report?**

We found that preschool-aged children with obesity in Head Start experienced significant summer weight gain.

**What are the implications for public health practice?**

As with school-aged children, increased focus on the summer is warranted for promoting healthy weight among children in Head Start.

## Objective

School-aged children experience more rapid weight gain during summer than the academic year (1). Previous studies on summer weight gain among preschoolers have yielded mixed results (2–4). Having a better understanding of when preschoolers gain excess weight is important; 13.7% of 2- to 5-year-old children in the United States already have obesity (5), putting them at increased risk for obesity and adverse health outcomes at later ages (6). In our study, we used longitudinal height and weight data from more than 2,000 children to test the hypothesis that preschool-aged children enrolled in Head Start experience accelerated summer weight gain.

## Methods

### Sample

From fall 2016 through fall 2018, 2,539 children enrolled in 16 Head Start programs serving Boston, Cambridge, and Somerville, Massachusetts. The 2,044 children we included in the main analysis for this observational, longitudinal study had a valid body mass index (BMI) measurement in their first fall of Head Start. BMI data cleaning protocols removed 6.2% of BMI measurements (supplemental information is available at https://dataverse.harvard.edu/dataset.xhtml?persistentId=doi:10.7910/DVN/EXMNNM).

Sample characteristics extracted from Head Start administrative records include child age, sex, race/ethnicity (Hispanic/Latino, non-Hispanic Asian, non-Hispanic Black or African American, non-Hispanic White, and non-Hispanic other or multiracial), and calendar year of Head Start entry, as well as parent age, sex, education (less than high school, high school graduate, greater than high school), and family structure (single parent or 2 parents). We used maternal characteristics when available; more than 96% of parents in the sample identified as mothers or mother figures.

### Modified BMI z-score

Each fall and spring, trained Head Start staff measure children’s height and weight by using standardized equipment provided for an ongoing obesity prevention trial (7). We used Centers for Disease Control and Prevention 2000 reference curves (8) to calculate age- and sex-specific weight status (underweight, healthy weight, overweight, obese).

As suggested elsewhere (9), we used modified BMI z-scores (mBMIz) as the study outcome; mBMIz is interpreted similarly to standard BMI z-scores (BMIz), with 0 indicating the median age- and sex-specific BMI. mBMIz is more appropriate for longitudinal analysis. Unlike BMIz, it is sensitive to change even for children with high BMIs. (Appendix 2 of the supplemental information contains more detail on mBMIz.)

### Analysis

We summarized sample characteristics at enrollment. We used continuous linear spline mixed models to estimate the rate of change in mBMIz during academic year 1, the summer, and academic year 2 by initial weight status. Random program-level intercepts, child-level intercepts, and child-level slopes were included to account for repeated measurements on children, who were nested in Head Start programs. (Appendix 3 of the supplemental information contains model specification details.) We fit models with maximum likelihood and used likelihood ratio tests for the null hypothesis that the rate of change in mBMIz was the same across year 1, summer, and year 2 of Head Start. The main analysis was unadjusted because initial weight status was weakly associated with the number of BMI measurements taken (*P* = .64). Alpha was set at .05 a priori. All analysis was completed in SAS version 9.4 (SAS Institute Inc). Institutional review board approval was granted by the Harvard T.H. Chan School of Public Health and Boston College.

## Results

In fall of their first year in Head Start, children were aged 29 to 47 months (Table). Most were Hispanic/Latino (45.5%) or non-Hispanic Black/African American (34.1%), and the prevalence of obesity was 18.4%.

**Table:** Characteristics of Children Enrolled in Head Start and Their Parents, Boston, Cambridge, and Somerville, Massachusetts, 2016–2019^a^

Characteristic	Number of BMI measurements				Total (N = 2,044)	*P* a
	1 (N = 266)	2 (N = 953)	3 (N = 199)	4 (N = 626)		
**Children**						
**Age, mo**						
29–33	13.9	10.9	13.6	13.4	12.3	<.001
34–38	42.9	35.9	38.2	39.0	38.0	
39–43	34.6	42.8	30.7	28.0	36.0	
44–47	8.7	10.4	17.6	19.7	13.7	
**Sex**						
Male	50.4	51.5	51.3	47.4	50.1	.45
Female	49.6	48.5	48.7	52.6	49.9	
**Race/ethnicity (missing, n = 8)**						
Non-Hispanic Asian	7.2	9.7	9.1	12.6	10.2	.04
Non-Hispanic Black/African American	31.4	33.7	31.2	37.0	34.1	
Hispanic/Latino	52.3	46.8	45.2	40.6	45.5	
Non-Hispanic other or multi-racial	2.7	3.6	5.0	4.5	3.9	
Non-Hispanic White	6.4	6.2	9.6	5.3	6.3	
**Calendar year of Head Start entry**						
2016	31.6	29.5	41.2	50.6	37.4	<.001
2017	25.2	24.6	58.8	49.4	35.6	
2018	43.2	46.0	0.0	0.0	27.1	
**Initial weight status**						
Underweight	6.4	3.3	4.0	3.8	3.9	.64
Healthy weight	60.5	62.6	64.8	63.1	62.7	
Overweight	14.3	14.9	14.6	15.7	15.0	
Obese	18.8	19.2	16.6	17.4	18.4	
**Parents**						
**Age, y (missing, n = 22)**						
17–25	21.1	13.5	18.1	13.9	15.0	.04
26–30	30.7	26.7	25.1	26.7	27.1	
31–35	21.5	27.6	29.7	25.3	26.3	
36–40	18.0	18.7	16.1	21.3	19.1	
>40	8.8	13.6	11.1	12.8	12.5	
**Sex (missing, n = 20)**						
Male	3.1	3.4	2.0	3.0	3.1	.78
Female	97.0	96.6	98.0	97.0	96.9	
**Education (missing, n = 73)**						
<High school	23.0	21.8	24.6	27.1	23.9	.15
High school graduate	36.2	39.9	39.0	40.0	39.4	
>High school	40.9	38.3	36.4	32.9	36.8	
**Family structure (missing, n = 1)**						
Single parent	69.8	61.8	65.3	62.9	63.5	.11
Two parents	30.2	38.2	34.7	37.1	36.5	

Abbreviation: BMI, body mass index.

a All sample characteristics are at the time of the child’s first fall semester in Head Start. *P* values determined by using χ^2^ test.

Overall, mBMIz trajectory changed significantly with the onset of summer (Figure, *P* < .001). Accelerated summer weight gain was most pronounced among children with obesity, who had an average change in mBMIz of −0.30/y during academic year 1, followed by a significant increase in rate of change to 1.05 mBMIz/y over the summer (*P* < .001), and a subsequent significant decrease in rate of change to −0.13 mBMIz/y during academic year 2 (*P* < .001). These results were robust to multivariable adjustment and stricter sample inclusion criteria (more information is available at https://dataverse.harvard.edu/dataset.xhtml?persistentId=doi:10.7910/DVN/EXMNNM). Accelerated summer weight gain was also observed among children with overweight, although the pattern was less pronounced and was not significant across all sensitivity analyses.

**Figure:**
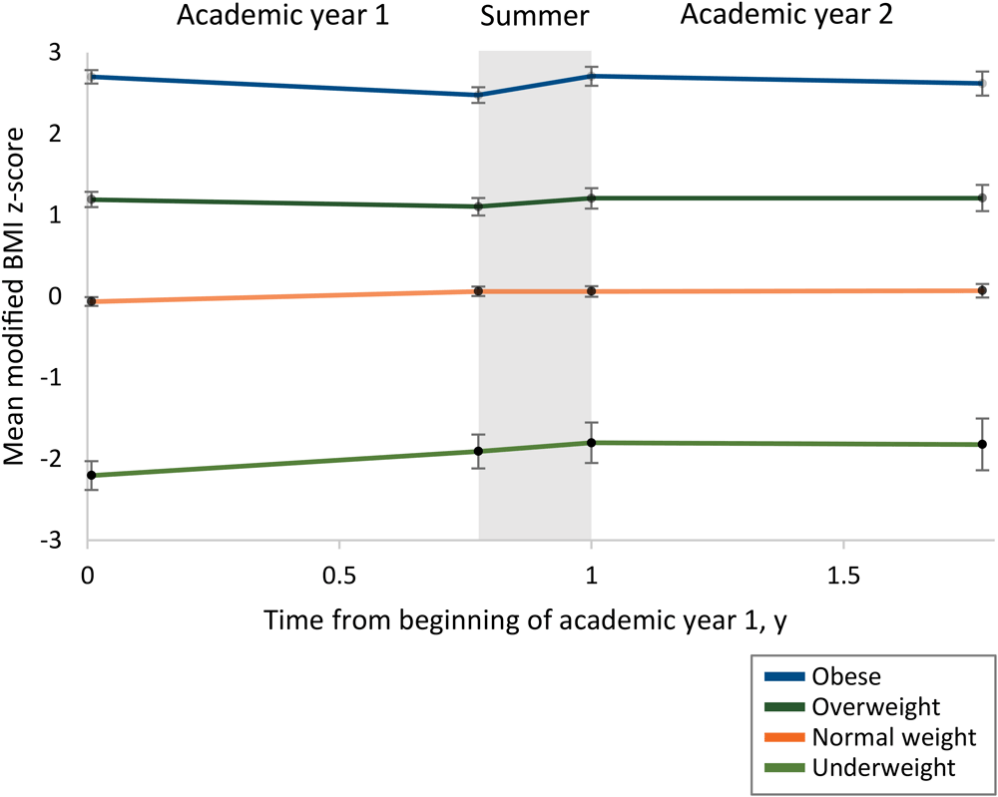
Mean modified body mass index (BMI) z-score among children who were underweight, healthy weight, overweight, and obese at enrollment in Head Start. Data are from children enrolled in Head Start in Boston, Cambridge, and Somerville, Massachusetts, 2016–2019. Estimates are from a single mixed-model with random program-level intercepts and random child-level intercepts and slopes. Error bars show 95% confidence intervals at the beginning of academic year 1, the end of academic year 1, the beginning of academic year 2, and the end of academic year 2. Across all children, mean modified BMI z-score trajectory changed significantly upon the onset of summer (*χ* ^2^ = 108.8, *df* = 15, *P* < .001).

**Table:** 

Weight Status	Time From Beginning of Academic Year 1, y	Mean Modified BMI z-Score (95% Confidence Interval)	*P* Value for Change in Slope From Previous Point
Underweight	0.01	–2.20 (–2.38 to –2.03)	NA
	0.78	–1.91 (–2.11 to –1.70)	NA
	1.00	–1.80 (–2.05 to –1.55)	.85
	1.78	–1.82 (–2.14 to –1.50)	.36
Healthy weight	0.01	–0.06 (–0.12 to –0.01)	NA
	0.78	0.06 (0 to 0.12)	NA
	1.00	0.06 (–0.01 to 0.13)	.20
	1.78	0.07 (–0.01 to 0.15)	.98
Overweight	0.01	1.19 (1.10 to 1.28)	NA
	0.78	1.10 (1.00 to 1.21)	NA
	1.00	1.21 (1.08 to 1.33)	.03
	1.78	1.21 (1.05 to 1.37)	.10
Obese	0.01	2.70 (2.62 to 2.78)	NA
	0.78	2.48 (2.38 to 2.57)	NA
	1.00	2.71 (2.59 to 2.83)	<.001
	1.78	2.62 (2.47 to 2.77)	<.001

## Discussion

Children in Head Start have distinct academic year and summer rates of change in mBMIz, and children with obesity experienced significantly accelerated summer weight gain. Similar patterns have been observed among school-aged children (1) and in 2 previous studies of preschoolers (2,4), adding to the evidence that efforts to promote healthy child weight may be most needed in summer. The lack of a structured daily schedule (10), the biological response to longer day length (11), or both may explain accelerated summer weight gain. However, more research is necessary among preschool-aged children, because another study found a decrease in BMIz over the summer among preschool-aged children with obesity (3).

Our study has many strengths that make it an important contribution to the literature. We used administrative BMI data for a large sample of preschool-aged children. Height and weight measurements were taken by trained Head Start staff members who used standardized equipment, and we used mBMIz for both longitudinal data cleaning and modeling. Previous studies that used BMIz rather than mBMIz may have underestimated the magnitude of changes for children with high BMI (9).

A potential limitation to this study is that, as in previous Head Start studies, many children had less than the maximum of 4 BMI measurements (2,3). We anticipate the magnitude of any selection bias induced by missing data to be small because weight status at enrollment was not significantly associated with the number of BMI measurements taken. Another limitation of this study is its generalizability, which was restricted to low-income children participating in Head Start. Patterns of weight gain may differ for higher-income preschool-aged children and for children in other early care and education settings, especially those not in center-based care during the academic year.

We found that preschool-aged children in Head Start with obesity experienced accelerated summer weight gain. Like school-aged children, increased focus on the summer is warranted for promoting healthy weight among children in Head Start.
